# Post-Mortem Grief Care for Family Caregivers After Home-Based End-of-Life Care: A Scoping Review

**DOI:** 10.3390/nursrep16030081

**Published:** 2026-02-26

**Authors:** Kazumi Hirano, Keiko Aizawa

**Affiliations:** 1 Department of Nursing, Faculty of Health Care and Medical Sports, Teikyo Heisei University, Ichihara 290-0193, Japan; 2 Department of Nursing, Faculty of Medical Sciences, Shonan University of Medical Sciences, Yokohama 231-0862, Japan; keiko.aizawa@sums.ac.jp

**Keywords:** bereavement care, early grief intervention, family caregivers, home-based end-of-life care, postmortem support, palliative home care, scoping review, community-based interventions

## Abstract

**Background/Objectives**: Evidence on postmortem grief care for family caregivers after home-based end-of-life care is limited. This scoping review aimed to map the content and effects of such interventions for adult family caregivers after home deaths. **Methods**: Following the Joanna Briggs Institute and Preferred Reporting Items for Systematic reviews and Meta-Analyses extension for Scoping Reviews guidelines, we searched PubMed, Cumulative Index to Nursing and Allied Health Literature, Embase, Cochrane Library, and Ichushi-Web from database inception to 31 March 2024. We included English- or Japanese-language intervention studies performed in home and community settings. “Early” grief care was defined as (i) support initiated within 6 months after the death of a loved one and (ii) interventions initiated during caregiving that assessed bereavement outcomes within 6 months after the death of a loved one. Data were charted and descriptively summarized. **Results**: From 4766 records, six studies were selected for the review (five randomized controlled trials and one ongoing registry trial). Interventions varied from dyadic psychological sessions integrated into specialist palliative home care (DOMUS) to brief psychoeducation, structured family-physician consultations, general-practice bereavement management with screening and stepped care, remote monitoring with nurse coaching during home hospice care, with bereavement outcomes assessed at 6 months (SCH), and an online self-help program for widowed older adults. The effects were mixed. DOMUS showed a small but significant reduction in caregiver anxiety; SCH reduced caregiver burden during caregiving and improved bereavement adjustment at 6 months. Other interventions did not demonstrate a clear advantage in outcomes over usual care. **Conclusions**: Early grief care after home-based end-of-life care is heterogeneous. Need-responsive multicomponent models embedded in existing home and community care pathways warrant further theory-informed evaluation.

## 1. Introduction

Grief is the sadness due to the death of a loved one; although grief often subsides over time, it can cause widespread psychological and physical effects. Bereavement is one of the most severe life stressors and adversely affects physical health, contributing to the worsening of underlying medical conditions, increased mortality, and elevated rates of suicide and depression, which may, in turn, disrupt daily functioning [1,2,3]. In grief, the risk of suicide is elevated, particularly within the first year following the loss [4]. When intense grief persists, individuals may be at risk of developing complicated grief, suggesting the need for professional support [5,6].

The World Health Organization (WHO)’s definition of palliative care clearly emphasizes the importance of bereavement support, stating that “7. Palliative care provides a support system to help families cope during the patient’s illness and in the aftermath of death,” and “8. Palliative care uses a team approach to address the needs of the patient and their family, including bereavement counseling, as needed” [7]. In the United States and the United Kingdom, bereavement support is often covered by insurance [8,9], and structured systems such as charitable organizations for bereaved individuals are relatively well established [10]. In contrast, in Japan, the aging population is associated with a high death rate, and the need for support for bereaved families has increased. In line with this, bereaved family care guidelines were published in 2022 [11]. However, most bereaved family care is not covered by public health insurance. Therefore, bereaved families rely on informal support from family members, and family structures have changed, leading to insufficient support.

Stroebe et al. defined grief as “an emotional reaction to loss that includes various psychological and physical symptoms” [12], with grief responses varying across individuals [13]. The loss of a loved one may evoke diverse emotions such as anger and guilt [14], and grief trajectories can change over time while differing across cultural contexts [15]. In terms of grief care, support for normal grief involves listening and empathy to the natural sadness, confusion, anger, and loneliness that occur after bereavement. However, bereaved family members at high risk require early support, and early detection through screening is important for this support. Furthermore, when grief is prolonged and causes significant disruption to daily life, specialized support for complicated grief and psychological difficulties may be required. In addition, support that is rooted in culture, religion, and the local community is important and respects the dignity and personal values of bereaved family members. Yamamoto defined grief care as support provided to help bereaved individuals come to terms with grief and experience gradual healing [16]. In this context, grief care involves offering emotional support to assist bereaved individuals in coping with their suffering [17]. Sakaguchi further emphasized that one of the most fundamental aspects of grief care is respecting the feelings of bereaved individuals and responding with gentle empathy. Additionally, the study identified the following main components of grief care: emotional support, instrumental support, informational support, and therapeutic intervention [18]. Emotional support encourages listening, empathy, and the expression of emotions. Instrumental support provides practical and lifestyle support, such as assistance with various procedures and reducing the burden of daily life; this is important because of the significant disruption in life immediately after bereavement. Informational support reduces excessive anxiety and misunderstanding by explaining that grieving is natural and providing information about the recovery process. Furthermore, medical and psychological professionals provide treatment as a therapeutic intervention if depression or anxiety becomes severe. Overall, grief care aims to support bereaved individuals in acknowledging their emotions, reconstructing their relationships with the deceased, and adapting to new ways of living [19].

However, family caregivers are already physically and mentally exhausted by the burden of caregiving, and this burden is likely to surface after the death of a loved one. In addition, they are more likely to be plagued by deep sadness, regret, and feelings of self-blame. Furthermore, caregiving often becomes the center of their lives, and they must adapt to the changes that follow the death of a loved one. A review by Vandersman et al. examining grief among family caregivers who had lost older relatives found that both the quality of end-of-life and post-death care influenced caregivers’ grief responses [20]. Similarly, a review by Nagraj et al. on grief care in primary health care settings demonstrated substantial variability in visit types and follow-up practices and suggested that family physicians and nurses are well positioned to provide responsive support to bereaved individuals [21]. However, post-bereavement grief care for families of patients who die following home-based care remains highly heterogeneous with respect to timing, duration, providers, target populations, settings, and delivery modalities. Given this variability, we considered a scoping review to be appropriate for comprehensively mapping existing evidence and characterizing intervention components and outcome effects.

Accordingly, this scoping review focused on bereaved family members of adult patients (aged ≥18 years) who received end-of-life care and died in community-based settings, including at home. This scoping review aimed to map the content and effects of early post-bereavement support interventions for family members and bereaved relatives following home- and community-based end-of-life care and to derive implications for effective early interventions after home death. This review is expected to lead to the establishment of intervention methods for early bereavement support for families after home-based end-of-life care, which is of great academic and social significance. A preliminary search of PubMed, Cumulative Index to Nursing and Allied Health Literature (CINAHL), Embase, Cochrane Library, and Ichushi-Web identified no existing systematic reviews or scoping reviews addressing this topic.

## 2. Materials and Methods

### 2.1. Review Framework

**Figure 1:**
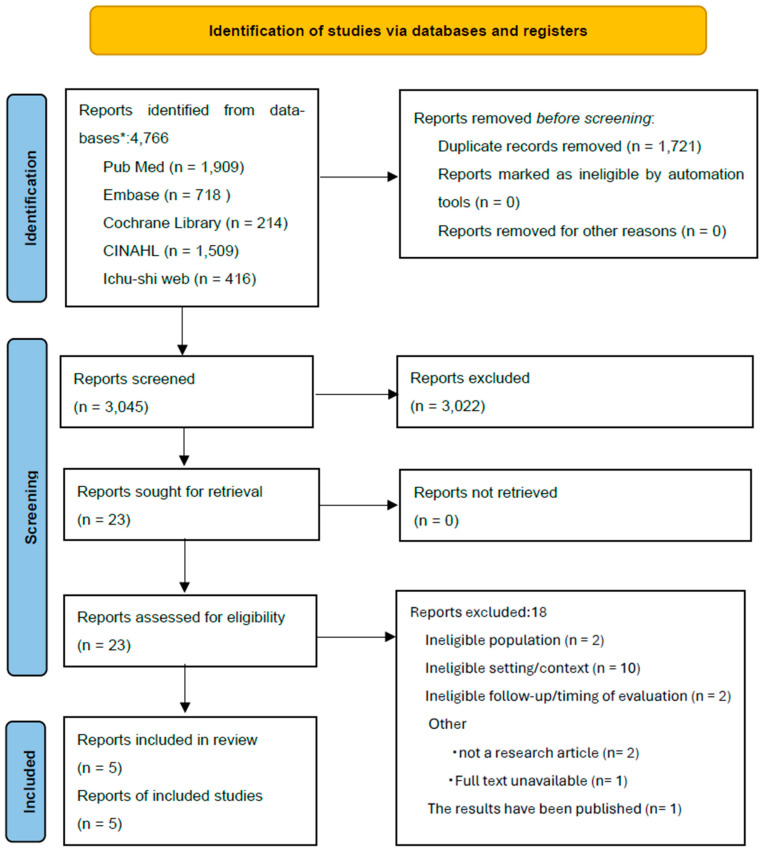
PRISMA 2020 flow diagram. * Databases searched: PubMed, Embase, Cochrane Library, CINAHL, and Ichu-shi Web.

This scoping review was conducted in accordance with the Joanna Briggs Institute Manual for Evidence Synthesis [22] and reported following the Preferred Reporting Items for Systematic reviews and Meta-Analyses extension for Scoping Reviews (PRISMA-ScR) [23]; the flow diagram is provided in Figure 1, and the reporting checklist is provided in Supplementary File S1. The review methods were specified a priori; however, the protocol was not registered. Study selection records and data extraction were managed using Microsoft Excel (version Microsoft Excel for Microsoft 365; Microsoft Corporation, Redmond, WA, USA). No equipment was sourced or used for this review.

#### 2.1.1. Population

We included family members and bereaved relatives aged ≥18 years who had experienced bereavement. Studies were eligible if bereavement support was delivered in home- or community-based contexts, including home hospice, home-based palliative care, primary care, community services, and online programs.

#### 2.1.2. Concept

Eligible interventions could be administered before or after the death of a loved one and were required to include a quantitative evaluation of intervention effects. Outcomes assessed in included studies could include grief-related measures and/or broader indicators of psychological adjustment (e.g., depression or anxiety), reflecting the diverse outcome approaches used in early bereavement support evaluations. At least one follow-up assessment within 24 months of bereavement was required. In this review, we defined “early” bereavement-related interventions as (i) support initiated within 6 months after the death of a loved one and (ii) interventions initiated during caregiving that assessed bereavement outcomes within 6 months after the death of a loved one. Outcome assessment time points varied across studies, with follow-up extending up to 24 months after loss.

Given the anticipated heterogeneity in intervention models and outcome measures, we planned a scoping approach focused on evidence mapping and descriptive synthesis.

#### 2.1.3. Context

This review focused on interventions implemented in home- and community-based settings.

### 2.2. Information Sources and Search Strategy

To comprehensively identify relevant literature, we searched five electronic databases: PubMed, CINAHL, Embase, Cochrane Library, and Ichushi-Web (Japan Medical Abstracts Society). Searches covered database inception through 31 March 2024. Publications in English and Japanese were eligible. Search strategy development and compilation of the initial record set (before deduplication) were conducted in collaboration with a medical librarian.

The search terms were developed using the Population–Concept–Context framework. The core strategy was as follows:

(bereavement OR grief OR mourning OR widow OR bereaved family OR death of parent OR death of spouse) AND (intervention OR support OR counseling OR therapy OR care)AND (home care OR home hospice OR community setting OR home death). 

Because the study aimed to map quantitatively and broadly evaluate bereavement-support interventions (including pragmatic and quasi-experimental evaluations), we did not apply study-design keywords/filters such as “trial” or “randomized” as mandatory search terms across databases to avoid loss of sensitivity due to inconsistent indexing. Intervention eligibility (i.e., an intervention with quantitative outcome evaluation) was determined during screening based on the predefined inclusion criteria. The full search strategies for all databases are provided in Appendix A (Appendix A.1, Appendix A.2, Appendix A.3, Appendix A.4 and Appendix A.5).

The search results were exported to Microsoft Excel for management, deduplication checks, and documentation of the screening process.

Citation searching (reference list screening) and handsearching of specific journals were not conducted.

### 2.3. Study Selection

All retrieved records were screened in two stages based on the predefined inclusion and exclusion criteria. First, the titles and abstracts were independently screened by two reviewers using Rayyan. Second, the full texts of potentially eligible articles were independently assessed by two reviewers to confirm eligibility. Discrepancies at either stage were resolved through discussion until consensus was reached. The selection process followed PRISMA-ScR recommendations.

Inclusion criteria

1. Family members and/or bereaved relatives aged ≥18 years.
2. Bereavement support delivered in home- or community-based settings (e.g., home hospice/home-based palliative care/primary care/community services/online).
3. This study quantitatively evaluated intervention effects and reported psychosocial outcomes relevant to bereavement support such as grief, adjustment, depression, and anxiety.
4. Interventions delivered by healthcare professionals or other specialized support providers.
5. Articles published in English or Japanese.
6. Outcome assessment included at least one time point within 24 months after bereavement.
7. Primarily original peer-reviewed research articles; protocols and public trial registrations were included when an intervention study was clearly specified.

Exclusion criteria

1. Studies including only participants aged <18 years.
2. Pre-loss-only interventions without reported post-bereavement outcomes.
3. Qualitative-only studies or studies without quantitative outcome evaluation.
4. Studies were conducted exclusively in hospital/institutional settings without clear home or community care positioning.

Studies meeting the inclusion criteria were included for data extraction and analysis.

### 2.4. Data Synthesis

We mapped intervention content and delivery features; grouped outcomes into domains (e.g., grief severity, bereavement adjustment, psychological symptoms, burden, health-care use); and synthesized the findings narratively and in tables (Table 1, Table 2 and Table 3: study characteristics and intervention summaries, outcome measures/timing and fidelity, and cross-study effect comparison by outcome domain). Quantitative results were extracted, as reported in the included studies; no additional statistical analyses were performed by the review team.

### 2.5. Data Extraction and Analysis

From the included studies, we extracted information on setting, timing and duration of support, intervention providers, participant characteristics, intervention content, fidelity, outcomes, and theoretical background. Data were extracted independently by two reviewers, with disagreements resolved through discussion to reach a consensus.

Extracted data were synthesized as an evidence map, organizing interventions across studies by (1) intervention components, (2) target recipients (e.g., individual vs. dyad; caregivers vs. widowed persons), (3) timing (pre-loss, early post-bereavement ≤6 months, and later follow-up), and (4) interventionists/providers and delivery systems.

## 3. Results

A total of 4766 records were identified (PubMed, n = 1909; Embase, n = 718; Cochrane Library, n = 214; CINAHL, n = 1509; Ichushi-Web, n = 416). After removing 1721 duplicates, 3045 records were screened by title. Of these, 57 underwent abstract screening, resulting in the exclusion of 34 records. Therefore, 23 full-text articles were assessed for eligibility. Following full-text review, five randomized controlled trials [RCTs] met the inclusion criteria and were included in the review.

### 3.1. Overview of the Included Studies

Table 1 summarizes study and intervention characteristics; Table 2 details outcome measures, assessment timing, and fidelity; and Table 3 provides a cross-study comparison of effects by outcome domain (completed trials only).

The five RCTs comprised two individually randomized trials [24,25], two cluster RCTs [28,29], and one multisite RCT [27]. In addition, one trial protocol/registration record (Portugal) with no posted results at the time of the search was identified [30]. It is mentioned here only to indicate ongoing research activity and was not included in the cross-study comparison of intervention effects. The studies were conducted in Denmark [24,29], Spain [28], Sweden [25], the United States [27], and Portugal (registry only) [30]. All studies targeted family members and/or bereaved relatives of adult patients who received home- or community-based end-of-life care, with outcomes assessed into bereavement. While most interventions were initiated during the early bereavement period, several began during caregiving and continued into bereavement [24,25,27]. Given the heterogeneity of intervention designs, outcomes, and delivery models, we synthesized the evidence as a component–target–timing–provider map to facilitate cross-study comparisons without assuming intervention equivalence (Table 1, Table 2 and Table 3).

### 3.2. Intervention Content

Across studies, interventions varied along two key dimensions: (1) *delivery modality* (face-to-face vs. remote/automated; individual vs. dyadic vs. group; primary care vs. specialist palliative care) and (2) *intervention components* (psychoeducation, supportive counseling, symptom/emotion monitoring, screening, and stepped care). To enable comparison across heterogeneous designs, we mapped interventions by components, target recipients, timing relative to the death (including early post-bereavement ≤6 months), and provider type.

The multisite Symptom Care at Home (SCH) [27] employed daily interactive voice response (IVR) check-ins during caregiving, with automated self-care coaching delivered immediately and nurse review within 48 h for high-severity alerts. The intervention functioned as a stepped escalation model embedded within usual hospice care and concluded at death; bereavement outcomes were assessed at 6 months after the death.

The DOMUS trial [24] integrated psychologist-led dyadic sessions into specialist palliative home care following hospital discharge. Two sessions were scheduled early after randomization (pre-loss), followed by monthly needs assessments, optional additional sessions, and one to two closing sessions post-loss. Content focused on education, coping, and family communication, grounded in an existential phenomenological framework emphasizing family reorganization.

A psychoeducational intervention in specialized palliative home care [25] delivered manual-based support tailored to caregiver needs for knowledge and support, informed by Andershed and Ternestedt’s theoretical framework [26].

Primary Bereavement Care (PBC) [28] delivered in primary care comprised structured consultations emphasizing emotional validation, psychoeducation, risk assessment, and referral when indicated. Family physicians completed extensive training and conducted monthly sessions, positioning the intervention within non-specific psychotherapy factors and continuity of care.

The Danish general practice bereavement management program [29] implemented a low-intensity, population-based model combining universal information provision with structured risk screening (e.g., ICG-R thresholds) and stepped follow-up for screen-positive individuals. Rather than relying on lengthy formal training, the program emphasizes feasibility through standardized materials and workflow integration.

The online self-help program LEAVES-PT [30] was a 10-week, self-guided online program for widowed older adults, consisting of 10 modules of evidence-based readings and exercises (approximately one module per week). The registry record states that the program is founded on a task model of mourning and the dual-process model of coping with bereavement. Optional remote support was not specified in the registry record.

### 3.3. Interventionists and Delivery Systems

The interventions were designed and delivered by nurses, clinical psychologists, family physicians, or interprofessional teams. SCH relied on hospice nurses for alert review and outreach [27]. DOMUS embedded clinical psychologists within specialist palliative care teams [24]. In primary care, PBC [28] and the Danish program [29] were primary care-led, and the psychoeducational intervention [25] was delivered by interprofessional teams including physicians, nurses, and social workers.

### 3.4. Dose and Timing of Intervention Delivery

Intervention timing and intensity varied substantially. SCH provided intensive, technology-enabled support during home hospice caregiving [27]. DOMUS spanned pre- and post-death phases with flexible session frequency [24]. The psychoeducational intervention delivered three sessions over 3 weeks during caregiving [25]. PBC comprised seven monthly sessions delivered during months 4–13 post-bereavement [28]. The Danish program emphasized screening and low-contact follow-up at 6 and 13 months post-loss [29].

### 3.5. Outcome Measures and Outcome Domains

Across studies, bereavement-related outcomes were commonly assessed; however, some trials primarily evaluated broader psychological outcomes such as anxiety and depression. The measures included grief severity (Texas Revised Inventory of Grief–II [TRIG-II] [25,28], TRIG at 6 months post-loss [25], Inventory of Complicated Grief–Revised [ICG-R] [29]), bereavement adjustment (assessed at 6 months post-loss among spouses/partners) [27], and the Attitudes to Grief Scale (AGS-13) [25]. Online registry-based intervention-planned grief (TRIG) was the primary outcome [29].

Among the included studies, some did not assess grief severity. These studies evaluated intervention effects primarily using broader psychological outcomes (e.g., anxiety or depression) as indicators of early post-bereavement adjustment.

Other key outcome domains included depression and anxiety (Symptom Checklist-92 [SCL-92] [24]; Symptom Checklist-90–Revised [SCL-90-R] [28]; Beck Depression Inventory–II [BDI-II] [29]; Hospital Anxiety and Depression Scale [HADS] [25]); health (Health Index [25]); quality of life (36-Item Short Form Health Survey [SF-36] [28]; and health-care utilization (number of visits and out-of-hours contacts) [29].

Assessment time points ranged from early bereavement to 24 months post-loss [24,25,26,27,28,29]. Given the substantial heterogeneity in measures and timing, quantitative synthesis was not performed.

Additionally, the substantial heterogeneity in outcome measures (e.g., TRIG/TRIG-II, ICG-R, AGS-13, HADS, and BDI-II) and assessment timing prevented meta-analysis; instead, we used this heterogeneity as a key finding and mapped how outcomes were assessed across intervention models.

### 3.6. Effects on Outcomes

SCH demonstrated significantly better bereavement adjustment at 6 months among spouses/partners in the intervention group (*p* < 0.007) [27]. In DOMUS, anxiety decreased significantly over time, whereas depression showed transient between-group differences at selected time points but not across the entire follow-up period [24].

No significant effects were observed in the psychoeducational intervention [25] or PBC [28]. The Danish program showed non-significant trends toward improvement in complicated grief (ICG-R) and reduced out-of-hours contacts; depressive symptoms decreased over time in both groups, with no statistically significant between-group differences reported [29]. Outcome data were unavailable for LEAVES-PT at the time of registry entry [30].

### 3.7. Content of Comparator Conditions (Usual Care)

Across the included trials, comparator conditions were grounded in standard care, as delivered in each country/setting, and designed to exclude intervention-specific active components.

In SCH [27], both groups received usual home hospice care and had access to standard hospice bereavement services after the patient died. The control condition combined usual hospice care with “reporting only” (daily check-in responses were collected, but automated coaching and nurse-initiated active outreach based on alerts were not provided).

In DOMUS [24], the control group received care as usual; referral to specialized palliative care could occur as needed (and 60% received specialized palliative care, on average 110 days later than the intervention group). However, manualized dyadic psychological sessions were not provided.

In PBC [28], the control group received usual family practice care. Although visit frequency was broadly matched between the groups (frequency matching), structured grief-focused consultations were not delivered.

In the Danish program [29], the control group received usual care, whereas the intervention group additionally received mailed bereavement information materials; these materials were not provided to the control group.

In the specialized psychoeducational intervention [25], the control group received standard support provided by home palliative care services; only the intervention group received invitations to attend the three-session intervention.

### 3.8. Risk Stratification and Screening

Approaches to identifying and managing risk varied across studies and could be categorized into four models: (a) screening based on scale cut-offs, (b) clinical judgment embedded within structured consultations, (c) alert-driven approaches based on symptom severity, and (d) universal interventions without risk screening.

The Danish general practice bereavement management program [29] used ICG-R cut-offs to identify high-risk bereaved individuals at 6 and 13 months post-loss, to identify individuals at higher risk and recommend stepped support, including additional consultations or referral to specialist services. In contrast, PBC [28] relied on family physicians’ ongoing clinical judgment during seven structured monthly consultations, allowing individualized assessment of grief- and depression-related signs and referral as needed. SCH [27] used an alert-driven approach during the caregiving phase, in which daily IVR self-monitoring triggered nurse review within 48 h for high-severity responses, enabling timely escalation of support. Although post-loss outcomes were assessed, this monitoring approach was not continued post-loss. In contrast, the psychoeducational intervention for family caregivers [25] adopted universal delivery models, enrolling participants based on caregiving or bereavement status rather than risk thresholds.

### 3.9. Adverse Events and Related Considerations

Across the included studies, no clear reports of serious adverse events attributable to the interventions were identified. Implementation processes and basic adherence were supported through mechanisms such as the 48 h alert review window in SCH [27], documented session delivery in PBC [28], and biweekly supervision in DOMUS [24]. In addition, the psychoeducational intervention study [25] reported the delivery of three manually guided sessions by an interprofessional team, indicating that some degree of standardization in intervention delivery was intended.

## 4. Discussion

In this review, we defined early grief care as (i) support initiated within 6 months after the death of a loved one and (ii) interventions initiated during caregiving that assessed bereavement outcomes within 6 months post-loss; additionally, we mapped the components and effects of support for adults bereaved after end-of-life care in non-hospital settings. Overall, interventions were diverse, and few demonstrated their clear or sustained effects. Consequently, the current evidence base remains insufficient to identify which components are effective, for whom, at what time points, and how.

Among the included studies, interventions showing more favorable effects tended to be multi-component and responsive to changing needs. The SCH model combined frequent remote monitoring with tailored supportive coaching, enabling timely adjustments to caregivers’ evolving symptoms [27]. Similarly, the DOMUS trial integrated structured follow-up within specialized palliative home care and extended support into bereavement [24]. Both interventions integrated assessment and monitoring with tailored responses and access to professional input rather than relying on a single modality. These features align with the view that early bereavement is characterized by rapid change and uncertainty, impeding the effectiveness of fixed, standardized approaches [31].

In the DOMUS trial [24], psychologists delivered structured sessions, treating patients and family caregivers as dyads and focusing on education, coping, and communication within the family unit. Anxiety decreased over the entire follow-up period, whereas depression showed time-specific but not sustained differences. Because bereaved individuals often report unmet needs related to including communication and practical support [32], a family-centered approach may help address needs during early bereavement [33]. Together, these findings suggest that continuity of support beginning during caregiving, coupled with flexible, need-responsive designs, may enhance effectiveness.

Interventions without demonstrated effects also provide important insight. An RCT of psychoeducational sessions during caregiving applied a clear theoretical framework but showed no between-group differences [25], indicating that theory-based manualization alone does not guarantee effectiveness. Bereavement-related outcomes are strongly influenced by time, social context, and baseline support, making short-term, single-component interventions difficult to evaluate. Similarly, evidence remains insufficient to establish long-term effects or effects on broader grief constructs, as illustrated by mixed anxiety and depression outcomes in DOMUS [24]. In addition, no effects were observed for PBC in primary care [28], a Danish general practice program [29], or the online self-help program LEAVES-PT [30]. Contributing factors likely include heterogeneity in outcome measures, assessment timing, and follow-up duration. However, statistical significance does not necessarily imply clinical relevance. Given the small number of trials and the heterogeneity in interventions and outcomes, even statistically significant effects should be interpreted cautiously, and the clinical applicability of the findings remains uncertain.

The implementation of interventions is strongly shaped by contextual factors such as the maturity of national health-care systems, prevailing cultural values, and the distribution of professional roles. In countries where primary care serves as the central point of coordination, general practitioners often function as the main providers of bereavement support, whereas in settings with well-developed community nursing, nurses frequently play a key role in early outreach. Moreover, cultural norms surrounding the expression of grief and the role of the family directly influence the acceptability of support and the adaptability of interventions. These international differences indicate that intervention models effective in one country cannot be assumed to transfer seamlessly to another; careful adaptation to each health-system and cultural context is essential.

Across the included studies, intervention delivery involved a range of providers (e.g., nurses, clinical psychologists, family physicians, and interprofessional teams) [24,25,26,27,28,29]. In SCH [27], for example, nurses delivered the caregiving-phase intervention. Japan has traditionally relied on culturally embedded grief care through rituals and kin and community networks. Furthermore, in Japan, bereavement rituals and family-centered values have traditionally provided informal support [34]. However, demographic changes, including family nuclearization and population aging, have increased the risk of social isolation among bereaved older adults [35,36,37]. Given evidence of increased suicide risk within 1 year of loss [4], effective grief care represents an urgent issue.

In Japan, professional bereavement support is provided by visiting nurses and other professionals through individual visits and telephone consultations, with no established system for interprofessional collaboration. However, Japanese visiting nurses often build close relationships with families during home-based end-of-life care, which may serve as a gateway for early support. Some evidence suggests that collaboration between primary care and general practice in providing support based on medical assessments may be beneficial [25,27]. These findings underscore the importance of interprofessional collaboration in Japan. The implementation of professional support may require operational adjustments, such as coordination of providers and alignment with ritual timing. Nonetheless, the principles identified in this review may apply to different cultures. Future studies should consider support models grounded in the continuity of relationships, leveraging the strengths of Japan’s visiting nurse system and incorporating options such as online consultations. Support should respect dignity and personal values while enabling bereaved individuals to continue daily life in ways consistent with themselves [38]. Providers familiar with families may be particularly well positioned to respond to diverse needs [39].

Bereavement support aims to alleviate grief and help bereaved individuals reconstruct their lives [40,41,42,43], while recognizing that many grief responses are normative and time-limited [15,44]. In essence, normal grief is a natural and normal reaction that everyone experiences after bereavement. However, grief significantly varies culturally and personally, and the boundary between “normal” and “abnormal” is unclear, making it difficult to judge immediately after bereavement. Providing unnecessary medical care or excessive intervention can undermine the dignity and autonomy of the bereaved. This ethical issue of “what is considered normal grief, and at what point should support be provided?” must be handled carefully. We believe it is important to create a system where anyone can receive the support they need when needed. This underscores the importance of screening to identify those who require targeted support. Prior relationships also matter: professionals involved before death may offer reassurance after bereavement [45]. Reviews by Vandersman et al. [20] and Nagraj et al. [21] similarly report variability in post-loss care and follow-up. Taken together, the findings of the present review suggest that combining multiple support elements and enabling early identification with timely outreach may be more effective than single-component approaches. These findings may offer implications for the future development, implementation, and evaluation of interventions in this field.

## 5. Limitations

Few studies have targeted the family members or bereaved relatives of adults who died after receiving palliative or end-of-life care in living settings outside hospitals; therefore, the implications for effective early interventions for families after home deaths are limited. Moreover, the evidence was derived from studies conducted in a few regions, limiting the ability to draw robust conclusions regarding effective interventions. Future research should conduct more detailed investigations of post-mortem grief care for families after home-based end-of-life care, considering different international cultural contexts. The key challenges include building on existing studies and accumulating evidence using diverse research methods while working with bereaved individuals.

Because the protocol was not publicly registered prior to the review, transparency was limited, and we cannot fully rule out potential duplication with ongoing or unpublished evidence syntheses. Furthermore, we did not conduct citation searching or handsearching; therefore, some relevant studies may have been missed despite the comprehensive multi-database search. Finally, Web of Science, Scopus, and PsycInfo were not searched; therefore, some relevant studies—particularly those indexed primarily in multidisciplinary or psychology-focused databases—may have been missed.

## 6. Implications for Nursing Practice

Based on the mapped evidence, post-mortem grief care after home-based end-of-life care may be strengthened through (1) proactive outreach to family caregivers after the patient’s death, (2) timely assessment of grief-related needs and risk, and (3) clear referral pathways to specialist bereavement support when indicated. There appears to be considerable variation across countries and regions in care providers’ bereavement-support knowledge and in the availability of support systems for families following home-based end-of-life care. The findings suggest a need for enhanced education for care providers regarding bereavement support, as well as the development of more robust support infrastructures.

## 7. Conclusions

This scoping review identified limited interventional evidence on early post-mortem bereavement support for family members after home-based end-of-life care. Interventions and outcomes varied widely, and grief severity was not consistently assessed, with several studies evaluating broader psychological adjustment instead. Future studies should test scalable, culturally sensitive interventions and adopt more consistent outcome frameworks with robust designs.

## Figures and Tables

**Table 1:** Summary of the included studies.

Study/Country/Design/Setting	Participants/Comparator/Timing	Intervention	Dose/Delivery (Provider; Format/Duration)	Primary OUTCOME(s)/Main Effect
[24] Denmark/Parallel RCT (dyads)/Home-based SPC service	P: Caregivers of patients with incurable cancer (patient–caregiver dyads)/C: Usual care (delayed SPC)/T: Pre-loss BL; FU ≤ 19 mo incl. early post-loss.	DOMUS: manualized dyadic psychological sessions integrated into rapid transition to home-based SPC; brief closing sessions soon after the death.	Median 4 pre-loss sessions (IQR 2–6); 0–1 early post-loss sessions; 63% delivered in true dyads.	Anxiety (SCL-92): overall β = −0.12 (95% CI −0.22 to −0.01), *p* = 0.0266. Depression (SCL-92): Overall, ns.
[25] Sweden/ Explorative longitudinal study (RCT-derived; 2-arm)/Specialized palliative home care (10 facilities)	P: Family caregivers (n = 117 completers; IG 58/CG 59)/C: Standard support/T: Baseline; post-intervention; 2-month FU; 6 months after patient death.	Manualized psychoeducational intervention (Andershed & Ternestedt’s framework [26]; targeting caregivers’ needs for “knowledge” and “support”).	3 sessions over 3 weeks; delivered by palliative-care professionals (physician, nurse, social worker/priest).	Pre-loss grief (AGS-13), post-death grief (TRIG), anxiety/depression (HADS), and health (HI). No significant between-group differences reported.
[27] USA/Multisite RCT/Home hospice cancer programs (13 community sites)	P: Family caregivers of adults in home hospice (n = 332 analyzed: 159 SCH/173 UC) C: Usual hospice + daily reporting/T: Weekly burden during caregiving; bereavement at 6 mo (spouse/partner).	(SCH: daily automated telephone (IVR) check-ins; tailored self-care coaching messages delivered immediately; high-severity alerts reviewed by hospice nurses ≤48 h with follow-up as needed.	Daily IVR check-ins; tailored coaching messages; nurse follow-up triggered by alerts.	Caregiver burden: mean impact across period MD = 9.32 points (95% CI 5.67–12.97), *p* < 0.001; week-8 ≈ 38% reduction (Cohen’s d ≈ 0.61). Bereavement adjustment at 6 mo: SCH > UC, *p* < 0.007.
[28] Spain/Cluster RCT (physicians)/Primary care family-practice clinics	P: Widows 4–13 mo post-loss; 31 FPs; analyzed 43 IG / 44 CG/C: Usual care (frequency-matched)/T: FU at 4/10/16/24 mo post-loss.	PBC: structured supportive consultations emphasizing listening/validation, “normalization” (psychoeducation on grief), risk screening, and referral if indicated.	FP training 56 h before delivery; ~45 min face-to-face monthly ×7.	Grief (TRIG-II): adjusted difference at 10 mo −2.90 (95% CI −5.92 to 0.12), *p* = 0.060 (not significant).
[29] Denmark/Cluster RCT (practice-level)/General practice (bereavement management program)	P: Bereaved relatives of deceased cancer patients recruited consecutively via participating hospitals/hospice services/C: Usual care/T: Questionnaires at 2, 6, and 13 mo post-loss.	GP bereavement management program: mailed information pamphlets to both GPs and bereaved relatives. GPs also received feedback on the relative’s baseline risk assessment based on depression at ~8 weeks post-loss.	Practice-based delivery via general practice; provider training not described.	Grief (ICG-R): decreased over time in both groups; no significant between-group differences. Health-care use: fewer out-of-hours GP contacts (IRR ≈ 0.55; 95% CI 0.29–1.06; *p* = 0.07).

AGS-13, Attitudes to Grief Scale (13 items); BDI-II, Beck Depression Inventory–II; BL, baseline; CG, control group; CI, confidence interval; FACT-G, Functional Assessment of Cancer Therapy–General; FP, family physician; FU, follow-up; GP, general practitioner; HADS, Hospital Anxiety and Depression Scale; HI, Health Index; ICG-R, Inventory of Complicated Grief–Revised; IG, intervention group; IRR, incidence rate ratio; IVR, interactive voice response; MD, mean difference; mo, month(s); ns, not significant; P, participants; PBC, Primary Bereavement Care; RCT, randomized controlled trial; SCH, Symptom Care at Home; SCL-90-R, Symptom Checklist-90–Revised; SCL-92, Symptom Checklist-92; SF-36, 36-Item Short Form Health Survey; SPC, specialist palliative care; T, timing; TRIG, Texas Revised Inventory of Grief; UC, usual care.

**Table 2:** Data collection timing, outcome measures, main effects, and fidelity.

Study	Data Collection Timing (Assessment Windows)	Outcome Measures (Primary Focus)	Main Effect (Concise)	Fidelity/Implementation Notes
[24]	Pre-loss baseline; follow-ups at 2/4/8 wk and 6 mo after randomization, and at 2 wk, 2/7/13/19 mo post-loss.	Anxiety and depression (SCL-92).	Anxiety reduced overall (β = −0.12; 95% CI −0.22 to −0.01; *p* = 0.0266). Depression overall ns; point-specific reductions observed at 8 wk and 6 mo after randomization, and at 2 wk and 2 mo post-loss.	Manualized content; psychologists with bi-weekly supervision; majority of sessions delivered as dyads; intervention adherence was not measured.
[25]	Baseline; FU1 & FU2 pre-loss; post-loss FU at 6 mo (timing of last FU varied depending on time of death).	AGS-13 (pre-loss only), TRIG (6-mo post-loss), HADS, HI.	No significant between-group differences in grief (TRIG I/II).	Manualized psychoeducation; 3 sessions over 3 wk; delivered by an interprofessional team.
[27]	Caregiving phase: weekly burden estimated from daily IVR reports across the hospice trajectory (modeled at wk 1 and 8); Bereavement: 6-mo assessment (spouse/partner subset).	Caregiver burden (weekly composite); bereavement adjustment at 6 mo.	Caregiver burden (weekly). Mean impact across period: MD = 9.32 pts (95% CI 5.67–12.97), *p* < 0.001; week-8 −38% (Cohen’s d = 0.61). 6-mo bereavement adjustment (spouse/partner): SCH > UC, *p* < 0.007.	IVR daily check-ins; alerts reviewed ≤48 h by nurses; outreach only on alert days (stepped-care operations).
[28]	Post-loss assessments at 4, 10, 16, and 24 mo; widows were identified ≤3 mo post-loss; baseline questionnaire administered at 4 mo.	Grief (TRIG-II) as primary; SF-36 domains (secondary).	TRIG-II at 10 mo: adjusted difference −2.90 (95% CI −5.92 to 0.12), *p* = 0.060 (ns). Selected SF-36 domains showed no intervention superiority overall.	FPs completed 56 h training; monthly 45 min sessions ×7; session logs ensured component delivery.
[29]	Baseline (~8 weeks/2 mo post-loss) depression-based risk assessment; questionnaires at 2, 6, and 13 mo post-loss.	Grief (ICG-R; 6 and 13 months), depression (BDI-II; 2, 6, and 13 mo), and health-care use (including out-of-hours GP contacts).	ICG-R mean: 6 mo IG 17.18 vs. CG 17.06; 13 mo IG 14.73 vs. CG 15.57. BDI-II mean: 2-mo (baseline) IG 11.46 vs. CG 12.22; 6 mo IG 9.23 vs. CG 10.19; 13 mo IG 7.85 vs. CG 8.84.	Mailed pamphlets to bereaved relatives and their GPs; GP feedback based on depression at ~8 wk post-loss; provider training not described.

AGS-13, Attitudes to Grief Scale–13; BDI-II, Beck Depression Inventory–II; CG, control group; CI, confidence interval; d, Cohen’s d; FP, family physician; FU, follow-up; HADS, Hospital Anxiety and Depression Scale; HI, Health Index; ICG-R, Inventory of Complicated Grief–Revised; IG, intervention group; IRR, incidence rate ratio; IVR, interactive voice response; MD, mean difference; mo, month(s); ns, not significant; *p*, *p*-value; RCT, randomized controlled trial; SCH, Symptom Care at Home; SCL-92, Symptom Checklist-92; SF-36, 36-Item Short Form Health Survey; SPC, specialist palliative care; TRIG, Texas Revised Inventory of Grief (TRIG I/II denote subscales); UC, usual care; w/wk, week(s); β, regression coefficient.

**Table 3:** Cross-study effect comparison by domain.

Study	Grief Severity	Anxiety	Depression	Caregiver Burden	Bereavement Adjustment (6 mo)	Health-Care Use	Overall Inference
[24]	Not assessed	Improved (β = −0.12; *p* = 0.0266)	Overall, ns; significant reductions at 8 wk and 6 mo post-randomization and at 2 wk and 2 mo post-loss.	Not assessed	Not assessed	Not assessed	Dyadic psychological support within SPC yielded a small but meaningful reduction in anxiety; depression not sustained overall.
[25]	No significant difference (TRIG I/II at 6-mo post-loss)	No significant difference (HADS-A)	No significant difference (HADS-D)	Not assessed	Not assessed	Not assessed	Manualized psychoeducation (3 sessions/3 weeks) in home palliative care: no between-group differences in grief/anxiety/depression (time effects observed for anxiety/depression).
[27]	Not assessed	Not a primary outcome (captured within burden subcomponents; Mood/Vitality improved, *p* < 0.001)	Not a primary outcome (captured within burden subcomponents; Depressed mood improved, *p* < 0.001)	Improved (MD = 9.32; *p* < 0.001; d ≈ 0.61 at wk-8)	Improved (spouse/partner; *p* < 0.007)	Not assessed	Digital stepped-care reduced burden during caregiving and improved 6-mo bereavement adjustment in spouses/partners.
[28]	No significant difference	Not assessed	Secondary only (ns overall)	Not assessed	Not assessed	Not assessed	FP-delivered structured consultations did not outperform usual care for TRIG-II; benefits not demonstrated, and some secondary outcomes favored control (e.g., SF-36 Emotional Role).
[29]	No significant between-group difference in change (ICG-R:P = 0.07)	Not assessed	No clear between-group difference reported (BDI-II)	Not assessed	Not assessed	No significant difference (out-of-hours IRR ≈ 0.55; *p* = 0.07) Fewer out-of-hours GP contacts (trend): IRR ≈ 0.55; *p* = 0.07	Practice-level screening/info showed near-significant improvements in grief and utilization, but not definitive.

AGS-13, Attitudes to Grief Scale–13; BDI-II, Beck Depression Inventory–II; CG, control group; FU, follow-up; FP, family physician; HADS, Hospital Anxiety and Depression Scale (HADS-A, anxiety subscale; HADS-D, depression subscale); HI, Health Index; ICG-R, Inventory of Complicated Grief–Revised; IG, intervention group; IRR, incidence rate ratio; IVR, interactive voice response; MD, mean difference; mo, months; ns, not significant; RCT, randomized controlled trial; SCH, Symptom Care at Home; SCL-92, Symptom Checklist-92; SPC, specialist palliative care; TRIG, Texas Revised Inventory of Grief (TRIG-I/II where applicable); wk, weeks.

## Data Availability

Raw data supporting the conclusions of this study will be made available by the authors upon request.

## Supplementary Materials

The following supporting information can be downloaded at: https://www.mdpi.com/article/10.3390/nursrep16030081/s1, File S1: PRISMA 2020 Checklist. Reference [46] is cited in the supplementary materials.

## Public Involvement Statement

No public involvement in any aspect of this research.

## Guidelines and Standards Statement

This manuscript was prepared according to the PRISMA-ScR guidelines. The PRISMA 2020 checklist was additionally consulted to cross-check reporting completeness, and the completed PRISMA 2020 checklist and flow diagram are provided.

## Abbreviations

The following abbreviations are used in this manuscript:

AGS-13	Attitudes to Grief Scale (13 items)
BDI-II	Beck Depression Inventory–II
CG	Control group
CI	Confidence interval
CINAHL	Cumulative Index to Nursing and Allied Health Literature
DOMUS	Dyadic psychological intervention integrated into specialist palliative home care
FU	Follow-up
GP	General practitioner
HADS	Hospital Anxiety and Depression Scale
HI	Health Index
ICG-R	Inventory of Complicated Grief–Revised
IG	Intervention group
IRR	Incidence rate ratio
IVR	Interactive voice response
JBI	Joanna Briggs Institute
MD	Mean difference
ns	Not significant
P	Participants
PBC	Primary Bereavement Care
PRISMA-ScR	Preferred Reporting Items for Systematic Reviews and Meta-Analyses extension for Scoping Reviews
RCT	Randomized controlled trial
SCH	Symptom Care at Home
SCL-90-R	Symptom Checklist–90–Revised
SCL-92	Symptom Checklist–92
SF-36	36-Item Short Form Health Survey
SPC	Specialist palliative care
TRIG	Texas Revised Inventory of Grief
UC	Usual care

## Appendix A

### Appendix A.1. Search Strategy (PubMed) (n = 1909), 19 March 2024

**Search**	**Query**	**Results**
#1	“bereavement”[MeSH Major Topic] OR “grief”[MeSH Terms] OR “grief care”[Title/Abstract:~2] OR “grieving care”[Title/Abstract:~2] OR “grief support”[Title/Abstract:~2] OR “grieving support”[Title/Abstract:~2] OR “grief supports”[Title/Abstract:~2] OR “grief supporting”[Title/Abstract:~2] OR “bereavement care”[Title/Abstract:~2] OR “bereaved care”[Title/Abstract:~2] OR “bereaved support”[Title/Abstract:~2] OR “bereavement support”[Title/Abstract:~2] OR “bereavement supports”[Title/Abstract:~2] OR “bereavement supporting”[Title/Abstract:~2] OR “postmortem care”[Title/Abstract:~2] OR “post mortem care”[Title/Abstract:~2] OR “after death care”[Title/Abstract:~2] OR “after death support”[Title/Abstract:~2] OR “after death supports”[Title/Abstract:~2] OR “after death supporting”[Title/Abstract:~2] OR “mourn*”[Title/Abstract]	16,467
#2	“community health services”[MeSH Terms] OR “nurses, community health”[MeSH Terms] OR “community health workers”[MeSH Terms] OR “nurse’s role”[MeSH Terms] OR “family practice”[MeSH Terms] OR “primary health care”[MeSH Terms] OR “home nursing”[MeSH Terms] OR “community health*”[Text Word] OR “general practi*”[Text Word] OR “family practi*”[Text Word] OR “primary health*”[Text Word] OR “community nurs*”[Text Word] OR “visiting nurs*”[Text Word] OR “district nurs*”[Text Word] OR “home care”[Title/Abstract:~2] OR “home healthcare”[Title/Abstract:~2] OR “home nursing”[Text Word] OR “community nursing”[Title/Abstract:~2] OR “community care”[Title/Abstract:~2] OR home[Text word]	964,293
#3	“family”[MeSH Terms] OR “home nursing”[MeSH Terms] OR “family nursing”[MeSH Terms] OR “Caregivers”[MeSH Terms] OR “family”[Text Word] OR “families”[Text Word] OR spous*[Text Word] OR “home nursing”[Text Word] OR “family nursing”[Text Word] OR “surviving family”[Title/Abstract:~2] OR “surviving families”[Title/Abstract:~2] OR home[Text word]	1,857,123
#4	#1 AND #2 AND #3	1909
Note: In PubMed, the asterisk (*) indicates truncation to retrieve word variants.		

### Appendix A.2. Search Strategy Embase (n = 718), 5 April 2024

**Search**	**Query**	**Results**
#1	‘bereavement support’/exp	1514
#2	‘grief’/exp	16,806
#3	((grief OR griev* OR bereave* OR postmortem OR ‘post mortem’ OR ‘after death’ OR ‘mourn*’) NEAR/2 (care OR support*)):ti,ab,kw	3333
#4	#1 OR #2 OR #3	19,450
#5	‘community care’/exp OR ‘community health nursing’/exp OR ‘general practice’/exp OR ‘primary health care’/exp OR ‘home care’/exp OR ‘visiting nursing service’/exp	505,458
#6	(((community OR primary OR home) NEAR/2 (health* OR care)):ti,ab,kw) OR (((general OR family) NEAR/2 practi*):ti,ab,kw) OR (((community OR visiting OR district) NEXT/2 nurs*):ti,ab,kw)	504,272
#7	#5 OR #6	751,850
#8	family’/exp OR ‘home care’/exp OR ‘family nursing’/exp OR ‘caregiver’/exp	812,633
#9	family:ti,ab,kw OR families:ti,ab,kw OR spous*:ti,ab,kw OR ‘home nurs*’:ti,ab,kw OR ‘family nurs*’:ti,ab,kw OR home:ti,ab,kw	1,884,250
#10	#8 OR #9	2,349,457
#11	#4 AND #7 AND #10	929
#12	#11 NOT ‘conference abstract’/it	718

### Appendix A.3. Search Strategy Cochrane Library (n = 214), 8 April 2024

**Search**	**Query**	**Results**
#1	[mh bereavement] OR [mh grief]	347
#2	(grieving OR grief OR bereav* OR postmortem OR post-mortem OR “after death” OR mourn*):ti,ab,kw	1702
#3	#1 OR #2	1702
#4	[mh “community health services”] OR [mh “nurses, community health”] OR [mh “community health workers”] OR [mh “nurse’s role”] OR [mh “family practice”] OR [mh “primary health care”] OR [mh “home nursing”]	32,265
#5	((community NEAR/1 health*) OR (general NEXT practi*) OR (family NEAR/1 practi*) OR (primary NEXT health*) OR (community NEAR/1 nurs*) OR (visiting NEXT nurs*) OR (district NEXT nurs*) OR (home NEXT care) OR (home NEXT healthcare) OR (home NEXT nursing) OR (community NEXT care) OR home):ti,ab,kw	86,743
#6	#4 OR #5	102,334
#7	[mh family] OR [mh “home nursing”] OR [mh “family nursing”] OR [mh Caregivers]	17,899
#8	(family OR families OR spous* OR “home nursing” OR home OR parent* OR child* OR orphan* OR relative* OR surviving):ti,ab,kw	392,226
#9	#7 OR #8	394,126
#10	#3 AND #6 AND #9	224
#11	#10 in Cochrane Reviews	10
#12	#10 in Trials	214

### Appendix A.4. Search CINAHL (n = 1509), 19 March 2024

**Search**	**Query**	**Results**
S1	(MH “Bereavement+”) OR (MH “Grief+”) OR (MH “Death Counseling”)	17,173
S2	TI ((grief OR grieving OR bereavement OR bereaved OR postmortem OR “post mortem” OR “after death” OR “mourn*”) N2 (care OR support*)) OR AB ((grief OR grieving OR bereavement OR bereaved OR postmortem OR “post mortem” OR “after death” OR “mourn*”) N2 (care OR support*))	2649
S3	S1 OR S2	17,940
S4	(MH “Community Health Nurses+”) OR (MH “Community Health Services+”) OR (MH “Community Health Nursing+”) OR (MH “Home Health Care+”) OR (MH “Community Health Workers”) OR (MH “Nursing Role”) OR (MH “Family Practice”) OR (MH “Primary Health Care”) OR (MH “Home Nursing”)	708,315
S5	TI ((community OR primary OR home) W1 (health* OR care)) OR AB ((community OR primary OR home) W1 (health* OR care))	158,700
S6	TI ((general OR family) W1 practi*) OR AB ((general OR family) W1 practi*)	40,424
S7	TI ((community OR visit* OR district OR home) W1 nurs*) OR AB ((community OR visit* OR district OR home) W1 nurs*)	17,078
S8	S4 OR S5 OR S6 OR S7	794,670
S9	(MH “Family+”) OR (MH “Family Services”) OR (MH “Family Nursing”) OR (MH “Family Nurses”) OR (MH “Caregivers”)	313,945
S10	TI (“family” OR “families” OR spous* OR “home nursing” OR “family nursing” OR home) OR AB (“family” OR “families” OR spous* OR “home nursing” OR “family nursing” OR home)	452,498
S11	S9 OR S10	646,094
S12	S3 AND S8 AND S11	1522
S13	S12 Limiters: Publication Type: Dissertation	13
S14	S12 NOT S13	1509
Note: In CINAHL, “+” indicates an exploded subject heading.		

### Appendix A.5. Search Ichu-Shi Web (n = 416), 12 March 2024

**Search**	**Query**	**Results**
#1	grief/TA OR Bereav/TA *(grief OR bereavement OR grief care)*	8091
#2	(community health services OR home visiting nursing OR visiting nurses OR community health workers OR nursing role OR primary health care OR home care/home setting)	465,362
#3	(family OR bereaved OR family caregivers OR home setting)	342,585
#4	#1 and #2 and #3	1735
#5	(#4) AND (PT ≠ Conference proceedings) *(exclude conference proceedings)*	1029
#6	(#4) AND (PT = Original articles, Reviews)	416
